# Holistic Care Clinic for People with Parkinson’s Disease: Outcome from a Newly Developed Service

**DOI:** 10.3390/brainsci16010043

**Published:** 2025-12-29

**Authors:** Lucia Ricciardi, Bryony Ishihara, Belen González-Herrero, Priyanka Pradhan, Alison Leake, Assunta Trinchillo, Monica Bernardo, Lucy Kerogoi, Patrice Gallogly, Dominic Paviour, Elena Makovac, Francesca Morgante

**Affiliations:** 1 Neuromodulation and Motor Control Section, Department of Psychology and Neuroscience, School of Health and Medical Sciences, City St. George’s, University of London, London SW17 0RE, UK; bryonyishihara@gmail.com (B.I.); alison.leake@stgeorges.nhs.uk (A.L.); assuntatrinchillo94@gmail.com (A.T.); d.paviour@nhs.net (D.P.); f.morgante@gmail.com (F.M.); 2 Departamento de Medicina, Universidad Autónoma de Barcelona (UAB), 08193 Bellaterra, Spain; 3 Queen’s Hospital, Barking, Havering and Redbridge University Hospitals, Romford RM7 0AG, UK; belen.gonzalezh@autonoma.cat (B.G.-H.); elena.makovac@brunel.ac.uk (E.M.); 4 Department of Adult Psychological Professions, St. George’s University Hospitals NHS Foundation Trust, London SW17 0QT, UK; drpriyankapradhan@gmail.com; 5 Neurology Department, St. George’s University Hospital, London SW17 0QT, UK; monica.antunesguerrabernardo@stgeorges.nhs.uk (M.B.); lucy.kerogoi@stgeorges.nhs.uk (L.K.); 6 Kingston Hospital, London KT2 7QB, UK; p.gallogly@nhs.net; 7 Department of Neurology, Brunel University London, Uxbridge UB8 3PH, UK; 8 Centre for Neuroimaging Science, Kings College London, London SE5 9RS, UK

**Keywords:** Parkinson’s disease, anxiety, non-motor symptoms, holistic care, wellness

## Abstract

**Background/Objectives**: Non-motor symptoms (NMS) in Parkinson’s disease (PD), particularly neuropsychiatric disturbances such as anxiety, significantly impact quality of life. The Holistic Care Clinic for Parkinson’s disease at St George’s Hospital offers multidisciplinary assessments and personalized care to address both motor and non-motor symptoms, aiming to improve patient well-being and empower patients to manage their health and enhance their quality of life. This study evaluated the effectiveness of a holistic management approach for PD patients with prominent non-motor symptoms, particularly neuropsychiatric issues, by analyzing clinical outcomes and patient feedback. **Methods**: A retrospective analysis was conducted on patients referred to the clinic between June 2022 and June 2023 for non-motor symptoms. Patients received comprehensive assessments, including clinical exams and interviews focused on neuropsychiatric symptoms, followed by individualized care plans. Interventions for anxiety included online psychoeducation and cardiac biofeedback. Outcomes were assessed using the Clinical Global Impression (CGI) scale and patient feedback on interventions. **Results**: Thirty patients (mean age 65.7 years, mean disease duration 7.8 years) were included. Anxiety was the primary referral reason (66%). CGI scores indicated that 62% of patients experienced improvement. Medications were adjusted in 14 patients and 65% improved. For anxiety, 13 patients attended the psychoeducation session, with 91% rating it “very likely”/”likely” to recommend. Ten patients completed cardiac biofeedback training, showing a significant reduction in Parkinson’s Anxiety Scale scores (*p* = 0.03), and 90% recommending it. **Conclusions**: The holistic care approach of PD patients resulted in significant improvements in clinical outcomes. Patient feedback indicates high satisfaction with the interventions, supporting their acceptability and overall satisfaction with the interventions.

## 1. Introduction

Health is defined by the World Health Organization (WHO) in 1964 as a state of complete physical, mental, and social well-being, not merely the absence of disease or infirmity. This holistic definition recognizes health as a multidimensional construct that includes physical, emotional, financial, social, and spiritual domains [1]. Holistic care integrates conventional and alternative treatments, lifestyle changes, and supportive interventions to address these wider determinants of health. The biopsychosocial model [2], proposed by George L. Engel, further emphasizes the interconnection of biological, psychological, and social factors in health, promoting patient-centered, multi-disciplinary care that improves outcomes, especially for chronic conditions.

Parkinson’s disease (PD) is a chronic neurodegenerative disorder with clinical manifestations encompassing motor and non-motor symptoms [3,4]. Up to 90% of individuals with PD experience at least one non-motor symptom, with common issues including depression, anxiety, sleep disturbances, and cognitive impairment [5]. The prevalence of these symptoms tends to increase with disease duration and severity; for instance, olfactory dysfunction and constipation can manifest early in the disease, while other symptoms like hallucinations and cognitive impairment often appear in later stages [5]. Neuropsychiatric symptoms like depression, anxiety, impulsive compulsive behaviours, apathy, and psychosis are common non-motor symptoms in people with PD (PwP), even in the early stages [6]. Though often overshadowed by motor symptoms, these issues significantly affect quality of life and daily functioning, increase caregiver burden, and raise the risk of nursing home admission [7,8].

The complex interplay between motor and nonmotor symptoms in PD highlights the need for specialized expertise and close interdisciplinary collaboration [6]. Evidence shows that non-pharmacological therapies effectively improve both symptom domains and enhance quality of life in PwP [9,10,11,12].

Multidisciplinary holistic care clinics have shown promising outcomes [13]. For example, in a randomized controlled trial, the IMPACT study reported improvements in activities of daily living and quality of life in PwP [14]. Although these effects were attenuated after adjustment for disease severity, the study nevertheless emphasized the potential value of integrated and individualized care models in PD management.

The International PD and Movement Disorders Society has recently created the Task Force on Parkinson’s Wellness and Holistic Health to promote and facilitate research and clinical guidance on holistic care of PwP [15].

Despite this evidence, most multidisciplinary or integrated-care models reported to date have been initiated via motor symptom burden or general rehabilitation needs. For example, the IMPACT study offered comprehensive care to PD patients but did not systematically recruit on the basis of non-motor/neuropsychiatric burden.

To address this gap, our group has established a Holistic Care Clinic (HCC) for PwP at St George’s Hospital (SGH) in London, United Kingdom, in June 2022. The clinic adopts a multidisciplinary, patient-centered approach that integrates the management of both motor and non-motor symptoms, with a particular emphasis on addressing complex neuropsychiatric disturbances.

In this context, the present work introduces a distinctive service model in which referral to a holistic care clinic is triggered primarily by bothersome non-motor and neuropsychiatric symptoms rather than by motor complications. By inverting the conventional referral paradigm, and placing non-motor distress at the forefront, we aim to address a critical and under-recognized dimension of PD that aligns closely with patients’ lived experiences. This “non-motor first” referral strategy represents a pragmatic shift and, we think, a novel contribution to the evolving landscape of PD care.

This manuscript is intended to share early implementation outcomes from this newly established HCC for PD, focusing on acceptability, feasibility, and patient-centered outcomes.

The practical significance lies in the potential to improve patient well-being and overall treatment outcomes through an integrated model of care that targets not only motor symptoms but also the psychological and social factors affecting PwP.

## 2. Materials and Methods

In this retrospective study, we analyzed the clinical outcomes in all consecutive patients referred to the multidisciplinary Holistic Care Clinic for PD at St George’s Hospital, London.

Referral Pathway: Patients were referred to the HCC through the St George’s Hospital Movement Disorders service, affiliated neurology clinics, or local district general hospitals, based on clinical need. Patients were referred if they presented with bothersome non-motor symptoms in the context of PD, with a particular focus on neuropsychiatric features such as anxiety, depression, apathy, impulse control disorders, sleep disturbances, or non-motor fluctuations.

Inclusion criteria were as follows: diagnosis of PD; presence of one or more distressing non-motor symptoms impacting quality of life; willingness and ability to engage in psychoeducation and lifestyle-based interventions.

Exclusion criteria were as follows: referrals based solely on motor symptom management without prominent non-motor burden; significant cognitive impairment limiting participation in therapeutic interventions; active severe psychiatric illness requiring urgent specialist input (e.g., acute psychosis or suicidality); patients already receiving coordinated multidisciplinary care for PD through other services.

The analysis covered the period from June 2022 to June 2023. The Holistic Care Clinic for PD is a multidisciplinary assessment service run monthly by a movement disorders consultant and a specialist PD nurse, and cases are regularly discussed with a neuropsychologist and a neuropsychiatrist.

### 2.1. Description of the Holistic Care Clinic for PwP

#### 2.1.1. Intervention

Each patient attending the clinic undergoes a comprehensive clinical examination led by the neurologist, who assesses both motor and non-motor aspects of PD. This evaluation includes a semi-structured interview specifically focused on identifying and appraising neuropsychiatric symptoms. In this comprehensive approach, clinic discussion extends beyond clinical symptoms to include an evaluation of the patient’s social history, diet, and physical exercise habits, and the effects of PD on family dynamics and social relationships.

Based on the interview and clinical examination, an individualized care plan is discussed with the patient and with their care-partner if present. This plan addresses the physical, emotional, and social challenges faced by patients, ultimately aiming to improve their quality of life and daily functioning. Details of the core interventions are shown in Table 1 and Supplementary Table S1.

**Figure 1:**
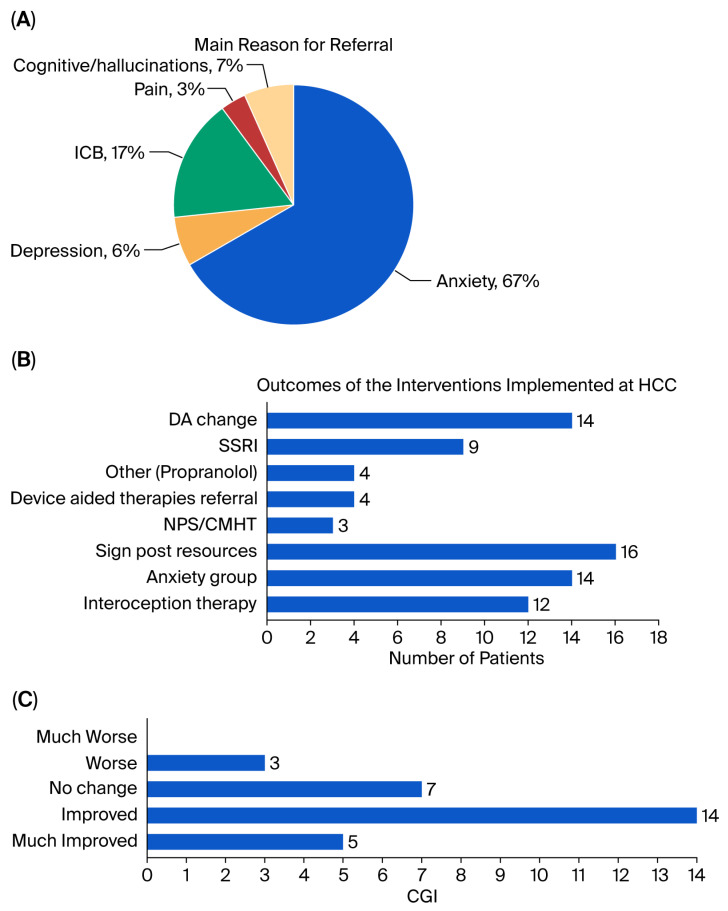
Overview of referral reasons, interventions, and clinical outcomes at the Holistic Care Clinic (HCC). (**A**) Primary reason for referral among the 30 patients assessed at HCC. Anxiety was the leading reason (67%), followed by impulse control behaviours (ICB), depression, pain, and cognitive symptoms or hallucinations. (**B**) Interventions implemented following multidisciplinary assessment. Patients could receive more than one intervention. (**C**) Clinical outcomes at follow-up, based on the Clinical Global Impression–Improvement (CGI-I) scale. Most patients were rated as "Improved" or "Much Improved", indicating positive clinician-assessed changes after intervention.

Interventions were grouped into the following categories: pharmacological changes, referrals to internal and external services, and lifestyle-focused advice. Patients often received multiple interventions based on clinical need. These categories were used for descriptive analysis, and their distribution is illustrated in Figure 1B. Follow-up assessment was conducted 3–4 months after the first assessment.

#### 2.1.2. Care Plan

The care plan may include various components tailored to the individual’s needs, including optimizing pharmacological therapies and providing mental health support to manage mood disorders or cognitive impairment. Patients may be directed to online self-management resources, including mindfulness and meditation tools, and referred to a clinical psychologist, neuropsychologist, or neuropsychiatrist as necessary. Sleep hygiene is discussed, and regular physical exercise is encouraged, with recommendations for local or online physical activity resources such as gym classes, dance, yoga, Nordic walking, or steady boxing. Referrals to neurophysiotherapy, occupational therapy, and speech therapy are made as needed.

Nutritional guidance is also a key part of the plan, with an emphasis on a balanced diet that supports neurological health, along with advice on hydration and managing common issues like constipation. The patient’s social history, family dynamics, and relationships are considered, ensuring the care plan addresses the broader impact of PD on their social and family life.

In addition, caregiver support and education are integral to the plan, helping to reduce caregiver burden and foster a collaborative approach to care that improves both patient and caregiver well-being.

#### 2.1.3. Interventions for Mental Health/Anxiety

As part of the treatment plan, we offer PwP with prominent anxiety 2 interventions developed by our team:

A. Psychoeducation online meeting. This is a 1.5-h online group meeting with the neuropsychologist and the neurologist aimed at giving information and strategies for the self-management of anxiety in PD. After being seen in the Holistic Care Clinic, patients with prominent anxiety are invited to attend an online group session called ‘Understanding and managing anxiety in PwP’. The patients are also encouraged to bring a spouse, partner, family member or friend if they so wish. The content and aims of the session are to provide brief information on how PD affects the brain, and explore the relationship between the symptoms of anxiety and PD and the reasons why anxiety may manifest in PD, ranging from neurophysiological, medication side effects and psychological reaction to living with reduced physical ability. The second half of the session then takes attendees through some psychological interventions rooted in Acceptance and Commitment Therapy that also incorporates body-based techniques such as grounding and coherent breathing that are evidence based for helping to manage anxiety [16]. Finally, elements of Compassion Focused Therapy are integrated into the psychoeducation programme, based on the recent evidence provided by our group on the positive effects of these practices on physiological recovery from stress, anxiety depression and PD-related stigma [17].

B. Cardiac biofeedback intervention: this was a one-to-one session led by the neurologist where patients were shown how their heart-rate changes in real time (cardiac biofeedback) during exposure to stress followed by deep breathing. The protocol elicited anxiety and it allowed individuals to observe how their physiological signals were affected in parallel (heart rate changes). Next, the patients were taught established and quick ways to self-sooth, or down regulate their anxiety and the corresponding physiological signals, while also providing them with direct visual feedback (biofeedback) of these changes and opportunities to reflect on their own ability to self-sooth. Patients were then invited to practice the deep breathing exercises at home and were provided with a video-guide for it.

This study was conducted in accordance with the principles of the Declaration of Helsinki. It is based on retrospective clinical data obtained during routine clinical care. All patient data were fully anonymized prior to analysis. The study was approved as a clinical audit by St George’s University Hospital NHS Foundation Trust (approval number: AUDI004189).

### 2.2. Outcome Measures

We explored the effect of the Holistic Care intervention using the Clinical Global Impression scale (CGI) [18].

The CGI was used to measure changes in symptoms at follow-up as compared to the baseline visit. This was completed by the neurologist at the follow-up consultation, after an intervention had been initiated. The following question was rated on a five-point scale: “Compared to the patient’s condition prior to the intervention initiation, this patient’s condition is: 1 = much improved since the initiation of treatment; 2 = improved; 3 = no change; 4 = worse; 5 = much worse; since the initiation of treatment” (modified from [19]).

The CGI scale was selected because of the retrospective design, which relied on clinicians’ documented impressions in the medical record.

For the cardiac biofeedback intervention, the severity of anxiety symptoms was assessed using the Parkinson’s anxiety scale (PAS) where a score > 13 indicates presence of anxiety.

Patient’s feedback forms were employed to evaluate the acceptability and overall satisfaction with the two interventions for anxiety that were offered, namely the psychoeducation online meeting and the cardiac biofeedback intervention. Following each session, patients were sent feedback forms asking: “How likely would you recommend this workshop/intervention to other people living with ‘Parkinson’s Disease and their relatives?”. Patients were requested to answer on a 5-point scale (Very likely/Likely/Neutral/Unlikely/Very unlikely).

Moreover, they were asked “Would you be interested in attending any further sessions” with a YES/NO answer.

### 2.3. Statistical Analysis

Descriptive statistics were used to summarize the demographic and clinical characteristics of the sample, as well as treatment outcomes. Categorical variables are reported as frequencies and percentages. Clinical outcomes were evaluated using the CGI scale, and the proportion of patients showing improvement was calculated.

Due to the ordinal structure of the CGI, ordinal regression was used to assess the relationship between CGI scores and clinical predictors (PD phenotype-akinetic rigid vs. tremor dominant- and disease duration), using a cumulative logit model. Model fit was evaluated with the likelihood ratio and Goodness-of-Fit tests; significance was set at *p* < 0.05. Ordinal regression accommodates non-normally distributed outcomes and is well-suited to assessing the cumulative effect of multiple clinical variables.

To measure the effect of the cardiac biofeedback intervention, a paired *t*-test compared PAS scores pre- and post-intervention. Analyses were performed in SPSS v30.0. The significance threshold was set at *p* < 0.05. Data are shown as mean ± standard deviation. The minimally clinically important difference score for the PAS was also calculated as ½ standard deviation of the baseline score [20].

## 3. Results

Twelve holistic clinics were run from June 2022 to June 2023 with a total of 30 patients (16 female, age: 65.7 ± 8.1 years, disease duration: 7.8 ± 4.6 years, levodopa equivalent daily dose: 567.8 ± 318.5).

The main reasons for referrals are outlined in Figure 1A. Anxiety was the most common reason for referrals (67%), followed by impulsive compulsive behaviour disorders (ICB). Referrals originated from neurologists (N = 14), PD nurses (N = 14) and general practitioners (N = 2).

The outcomes implemented at HCC are shown in Figure 1B. These included changes in dopaminergic medications in 14 patients (37%). Of these, 10 patients received an increase in dopaminergic treatment (levodopa in 9; dopamine agonists in 5) to address non-motor fluctuations, particularly anxiety symptoms emerging during wearing-off periods. In 4 patients, dopamine agonists were reduced due to bothersome visual hallucinations or impulse control disorders.

In 30% of the total sample (all of them presenting with anxiety except one who had moderate depression), either selective serotonin reuptake inhibitors (SSRIs) or serotonin and norepinephrine reuptake inhibitors (SNRIs) were prescribed, and the dose was increased, or it was changed to another agent. Finally, in 4 patients, a beta-blocker (propranolol) was prescribed to treat anxiety. All of these patients also had resting tremor as their main bothersome symptom.

Four patients (13.3%) were referred to the Advanced Therapies Movement Disorders Clinic and received apomorphine subcutaneous infusion (N = 3), Deep Brain Stimulation (N = 1) or an adjustment in the levodopa–carbidopa intestinal gel infusion.

Actions taken in PwP with anxiety (N = 8, 26.6%) and moderate depression (N = 1, 3.3%) included a new prescription or dose increase of antidepressants (selective serotonin reuptake inhibitors or serotonin and norepinephrine reuptake inhibitors) or a change to another agent. In four additional patients with anxiety, propranolol was prescribed. Three PwP who were referred for severe anxiety and comorbid psychosis were referred to the community mental health service after discussing with the HCC neuropsychiatrist. In two of these PwP, the diagnosis was revised to Lewy Body Dementia.

The effect of the overall holistic care plan was analyzed using the CGI in 29 PwP, as one subject was lost to follow-up. As per CGI scores, 64% of PwP improved significantly (17% ‘much better’, 47% ‘better’), 23% remained unchanged, and 10% worsened (Figure 1C).

The ordinal regression model indicated a significant overall model fit (chi-square = 62.9, *p* < 0.0001), suggesting that the independent variables (disease duration and clinical subtype) collectively provide a statistically significant explanation of variance in the CGI scores. However, individual predictors did not significantly contribute to the model (*p*-values > 0.05), indicating that it had no significant effect on the CGI.

Thirteen PwP participated in the online psychoeducational group and provided feedback, while one was unable to attend. When asked, “How likely would you recommend this workshop to other patients with PD and their relatives?”, 50% of participants responded with “very likely”, 41% “likely”, and one patient answered “neutral”.

Twelve PwP with anxiety were referred to the cardiac biofeedback intervention. Ten completed the training. Anxiety significantly improved after this intervention (PAS pre-intervention = 21.1 ± 9.1, post-intervention = 18.3 ± 6.1, *p* = 0.03). The mean score change was −2.8 ± 3.6. A total of 30% of participants (3/10) showed a clinically important difference (more than −4.6 points) in the PAS post-intervention [20]. To the question: “How likely would you recommend this session to other patients living with PD and anxiety”, 90% replied “very likely” or “likely”, and 1 PwP replied “neutral”.

## 4. Discussion

This study highlights the feasibility, acceptability and potential value of a Holistic Care Clinic (HCC) for people with Parkinson’s disease (PwP) with prominent non-motor symptoms, with 64% of patients showing improvement on the Clinical Global Impression (CGI) scale.

Our findings, even as preliminary pilot data, underscore the potential value of a care model anchored around non-motor symptom burden. By selecting patients on the basis of prominent neuropsychiatric and non-motor complaints, we reprioritized what triggers referral to specialized, multidisciplinary care.

Our findings reflect the benefits of individualized, multidisciplinary care focused on the complex neuropsychiatric and psychosocial dimensions of PD. The importance of treating non-motor symptoms is increasingly recognized, as neuropsychiatric manifestations such as anxiety and depression affect more than one-third of PwP and often have a greater negative impact on quality of life than motor impairment [4,5,6,7]. Addressing these symptoms requires a comprehensive, patient-centered approach integrating multiple domains of care [11,14].

Previous studies have reported variable outcomes for integrated care models in PD. For instance, the IMPACT study demonstrated improvements in activities of daily living and quality of life, but these effects were attenuated after adjustment for disease severity, highlighting the need to tailor interventions to individual patient needs [14].

Building upon this evidence, our HCC emphasizes individualized care through coordinated collaboration between a movement disorder neurologist, a Parkinson’s disease nurse specialist (PDNS), and allied health professionals. While “holistic” and “multidisciplinary” are often used interchangeably, our model extends beyond discipline-specific input by integrating mental health, social, and rehabilitative components into a unified care plan.

The PDNS played a pivotal role in coordinating care and supporting patient self-management, fulfilling a key priority of PwP to have a single, accessible point of contact [21,22]. The PDNS also facilitated treatment adjustments and promoted adherence, reinforcing the importance of specialized nursing input in holistic models [23,24]. In line with the framework of holistic healthcare, mental health was a central focus of the clinic, with anxiety identified as the most common reason for referral (67%). The management of anxiety in PD remains a major unmet need, as no randomized controlled trials (RCTs) have yet evaluated its treatment as a primary outcome [25]. Current pharmacological strategies, including SSRIs, provide only partial relief, underscoring the importance of integrating psychological therapies, physical activity, and psychosocial interventions in PD management [26,27].

To address this gap, our clinic implemented a mindfulness and cardiac biofeedback program designed to improve anxiety. This body–mind program targets the interaction between bodily sensations and emotional experiences, which has been shown to improve various clinical symptoms, including anxiety, across neuropsychiatric disorders. Good clinical outcomes from cardiac biofeedback are often linked to enhanced interoceptive awareness, suggesting this as a potential mechanism underlying symptom improvement [28]. Enhanced interoceptive awareness refers to an improved ability to perceive and understand internal bodily signals, like heart rate and breathing. Strengthening this awareness supports better regulation of emotional and stress responses. In anxiety, improving interoceptive awareness via cardiac biofeedback can lead to better anxiety symptom control by helping people to recognize and manage their physical reactions to stress [29].

Patient feedback underscored the acceptability and practicality of this intervention, particularly its ease of integration into daily routines. While this feedback captures satisfaction and perceived utility rather than formal efficacy, it supports the feasibility of incorporating such interventions into routine care.

In addition to medical and psychological therapies, psychoeducation remains a key component of our approach, providing PwP and caregivers with information and coping strategies to enhance understanding, self-efficacy, and engagement in care [30,31]. These educational sessions promote self-management practices such as regular exercise, sleep hygiene, and stress reduction—which align with growing evidence that lifestyle interventions combining physical activity, nutrition, and stress management may improve symptoms and possibly slow disease progression [32].

This pilot study has several limitations. The small sample size (N = 30) limits statistical power and generalizability, and the retrospective design restricts the ability to draw causal inferences. The CGI, although validated and widely used, offers a broad measure of improvement and may not fully capture the impact of specific interventions. Additionally, feedback forms assessed satisfaction rather than objective outcomes, and the absence of a control group precludes direct comparison with standard care. Confounding factors such as disease severity and comorbidities will also need to be systematically controlled in the future. Future prospective studies with larger samples and validated non-motor outcome measures are warranted to confirm these preliminary findings.

Future research should consider integrating objective digital measures, such as wearable- or app-based symptom tracking, to complement self-reported data and provide more continuous, ecologically valid insights into symptom fluctuations. Additionally, incorporating more structured neuropsychiatric assessments could help refine diagnostic precision and strengthen the characterization of symptom profiles. Such methodological enhancements may improve the robustness of findings and support the development of more personalized intervention strategies.

Despite the above limitations, the HCC proved feasible within an NHS framework, was well received by both patients and caregivers, and demonstrated potential for scalability across district general hospitals (DGHs).

## 5. Conclusions

We have described our experience of establishing and delivering a Holistic Care Clinic for PwP, designed to optimize management of complex non-motor and neuropsychiatric symptoms through an integrated, patient-centered approach. Our findings demonstrate notable improvements in patient outcomes with 64% of patients reporting a significant improvement in their condition on the Clinical Global Impression scores.

By combining neurological, psychological, and educational interventions with specialist nursing coordination, the clinic provides comprehensive care addressing both physical and mental health needs.

Moreover, our model emphasizes holistic assessment, encompassing motor and non-motor domains, but crucially starts from a different clinical entry point. This reorientation has implication not only for patient centered care, but for resource planning, interdisciplinary collaboration, and long-term service design. While further hypothesis-driven studies with larger, controlled and prospective designs will be required to establish efficacy, the present work demonstrates the feasibility and acceptability of a “non-motor first” holistic clinic.

In doing so, it challenges the field to reconsider current referral and care paradigms: rather than motor dysfunction dictating access to comprehensive care, the often neglected, yet highly disabling, neuropsychiatric and non-motor burden may serve as a valid and important referral trigger. We believe this shift has the potential to improve quality of life and fulfil unmet clinical needs among people living with PD.

## Figures and Tables

**Table 1:** Core Interventions in the Holistic Care Clinic for Parkinson’s Disease.

Domain	Intervention	Resources
PD Medication Adjustment	Individualized review and optimization of PD medications	Medication charts; patient-friendly leaflets explaining drug timing.
Mental Health Psychoeducation	Individualized review and psychoeducation focusing on anxiety, depression, apathy, and impulsivity. Referral to Mental Health Team if needed.	Printed/online resources; one-on-one explanation; online group meetings facilitated by neurologist and psychologist.
Anxiety Management	Referral to: ––Mindfulness with cardiac biofeedback program —Psychoeducation online group for anxiety.	Video guides for breathing/mindfulness; access to online and in-person group sessions; written materials; signposting to apps.
Physical Activity	Recommendation for regular activity based on patient ability: e.g., brisk walking, gym use, home exercises. Referral to physiotherapy team if needed.	Exercise handouts; referral to PD-specific classes (e.g., PD Warrior, dance, yoga); signposting to local opportunities.
Dietary Guidance	Tailored discussion covering fiber intake, fruit/veg consumption, food tracking (via App), and hydration. Constipation addressed proactively.	App suggestion for diet tracking; hydration and bowel health leaflets.
Sleep Hygiene	Advice on establishing sleep routines, managing nocturia, screen exposure, and bedtime practices.	Online and printed sleep hygiene information; relaxation App suggestions; clinician discussion on sleep–wake patterns.
Information & Signposting	All domains supported by printed or online educational materials. Patients are signposted to local and online resource.	Resources include PD choir, yoga classes, community dance, patient associations, support groups, and webinars.

## Data Availability

The data presented in this study are available on request from the corresponding author due to ethical restrictions.

## Supplementary Materials

The following supporting information can be downloaded at: https://www.mdpi.com/article/10.3390/brainsci16010043/s1. Table S1: Sustainability and Reproducibility Measures of the Holistic Care Clinic Model.
