# Supplementary material for: Holistic Care Clinic for People with Parkinson’s Disease: Outcome from a Newly Developed Service
Source: Brain Sci. 2025 Dec 29;16(1):43. doi: 10.3390/brainsci16010043 (PMC12839184; doi:10.3390/brainsci16010043)
Supplement: Supplementary file 1 [file brainsci-16-00043-s001.zip › brainsci-4000433-supplementary.pdf]

Supplementary Table S1: Sustainability and Reproducibility Measures of the Holistic Care Clinic Model

Domain	Component	Details
**Staffing**	Interdisciplinary team	Neurology, psychology, PD nurse collaborate in case planning and follow-up. Neuropsychiatrist, physiotherapist and dietician available for ad hoc referral.
**Training**	Initial team member training	1-day induction workshop covering holistic care planning, communication strategies, and lifestyle-based interventions.
	Ongoing support	Quarterly reflective practice MDT.
	Patient resource training	Clinical team members trained to provide standardized self-management tools, e.g., breathing exercise videos, food App, leaflets.
**Clinical Tools**	Standardised care templates	Structured holistic care templates used across all consultations to ensure consistency in documentation and follow-up planning.
	Shared digital resources	A digital repository of printable and video resources is used to ensure consistent patient education across team members.
**Scalability**	Manualized protocols	A detailed manual (in development) outlines protocols for replication, including case selection, intervention mapping, and outcome tracking.
**Evaluation**	Longitudinal patient tracking	Use of repeated measures (e.g., medication adjustments, symptom scales, patient-reported outcomes) to evaluate ongoing effectiveness.
	Feedback loop	Patient and clinician feedback is used iteratively to refine tools and delivery, supporting adaptive sustainability.
